# Development and implementation of a unified curriculum in math, statistics, computing, and informatics with financial considerations for medical physics graduate programs

**DOI:** 10.1002/acm2.70592

**Published:** 2026-05-14

**Authors:** Jenghwa Chang, Marissa Joyce Vaccarelli

**Affiliations:** ^1^ MS in Medical Physics Program, Department of Physics and Astronomy Hofstra University Hempstead New York USA; ^2^ Northwell New Hyde Park New York USA; ^3^ Donald and Barbara Zucker School of Medicine at Hofstra/Northwell Hempstead New York USA

**Keywords:** computational methods, course development, mathematical and statistical methods, medical informatics

## Abstract

**Background:**

AAPM Report No. 365 recommends that medical physics graduate programs offer courses covering both mathematical and statistical methods (Section 3.1.7) as well as computational methods and medical informatics (Section 3.1.8). While our program seeks to incorporate both of these essential areas into the curriculum, various financial and programmatic constraints have necessitated a more streamlined approach. Accordingly, this work presents a single 2‐semester‐hour course designed to address these topics in an integrated format.

**Purpose:**

In this paper, we present our efforts in developing a new teaching approach that addresses both AAPM Report No. 365 recommendations in one course.

**Methods:**

The major challenge of designing this course was the insufficient number of semester hours allocated to teaching both topics. To overcome this obstacle, we implemented a novel approach to homework assignments. Unlike the traditional approach in which students complete homework manually, students in this class were asked to write computer programs to solve most homework questions. These carefully designed assessments not only enhanced students’ understanding of the course materials but also required them to utilize appropriate computational methods. Recognizing that students had varying levels of coding experience from their undergraduate studies, the program instructed them, prior to starting the program, to acquire foundational Python skills through self‐guided learning to prepare for this course. In addition, basic Python programming guidance was provided with each homework assignment to support students with less coding experience.

**Results:**

The final course covered the following key mathematical concepts: signals and systems, Fourier series and transform, probability, statistical inference, image quality, optimization methods, and an introduction to artificial intelligence with an emphasis on machine learning. To complete the homework assignments, students developed coding skills in data visualization, numerical integration, convolution, continuous‐time/discrete‐time/fast Fourier transforms, random number generator, point estimation, confidence interval, hypothesis testing, linear models, DICOM, the Rose model, conjugate‐gradient descent, iterative methods for solving systems of linear equations, and support vector machines. The course was offered in the Spring 2024 and Spring 2025 semesters and received generally positive evaluations, with some noted challenges per the Course and Teacher Rating reports. Overall, students reported that the course was educationally beneficial; however, some indicated that the coding‐based assignments were demanding.

**Conclusion:**

We successfully developed and implemented a course that covers mathematical and statistical methods as well as computational methods and medical informatics as recommended in AAPM Report No. 365.

## LEARNING OBJECTIVES

In this paper, we present an innovative teaching method that addresses a key challenge in medical physics education: the limited semester hours available for instruction despite the expanding knowledge base required in the field. As a practical case study, we describe our implementation of the recommendations in AAPM Report No. 365^1^ under these constraints. Specifically, in the Spring 2024 semester, we developed and launched a new course that integrates the recommended mathematical and statistical methods with computational methods and medical informatics into a single curriculum, without increasing students' financial burden.

After reading this paper, readers will be able to:

1. **Recognize** the practical limitations encountered by our medical physics graduate program in developing a new curriculum aimed at improving program quality.
2. **Understand** the novel approach employed to address these limitations and design the new course.
3. **Adapt and apply** similar strategies when designing new courses within their own programs under comparable constraints.

## 1 INTRODUCTION

AAPM Report No. 365^1^ recommends that graduate medical physics programs include two topics: (1) Instruction in mathematical and statistical methods (Section 3.1.7), and (2) Computational methods and medical informatics (Section 3.1.8). Although these topics are listed under “Section 3.1 Core Topics,” they are not part of the “core graduate curriculum” required by CAMPEP^2^. As a result, graduate programs are not required to offer dedicated courses on these subjects. However, most programs strive to incorporate as much relevant material as possible to address these recommended topics on an impactful level.

At our institution, one natural solution would have been to ask our students to take additional courses within the computer science and mathematics departments. This approach is common among medical physics graduate programs, based on our experience reviewing applications for the medical physics residency program at our affiliated clinical institution.

In our view, this arrangement has several limitations. First, traditional computational methods courses offered by computer science departments often do not cover the domain‐specific knowledge needed for medical physics. Similarly, graduate‐level courses in mathematical and statistical methods tend to be highly theoretical and lack practical applications relevant to clinical work. Moreover, either approach limits the ability to tailor the curriculum specifically to medical physicists in training, due to factors such as discipline‐specific content needs, instructor expertise, and the relatively small enrollment typical of medical physics programs. Finally, requiring students to take these additional courses increases their credit load and, consequently, their financial burden.

We therefore decided to develop a dedicated course that addresses both recommended topics, focusing on the practical needs of clinical medical physics while minimizing students’ financial burden. The Master of Science in Medical Physics program at our institution is a 39–semester‐hour (39‐SH) degree designed to be completed over two years of full‐time study. The program includes eleven didactic courses, seven of which are designated as CAMPEP core courses, along with three practicum courses corresponding to each subspecialty within the field. In addition, students have opportunities to participate in basic, applied, and clinical research in medical physics.

Although we had the teaching capacity at both the university and program levels to offer multiple courses addressing the topics recommended in AAPM Report No. 365^1^, we sought to maintain the existing 39‐SH graduation requirement whenever possible in order to avoid increasing students’ financial burden. To achieve this, we needed to create space for the new course within the existing program structure. We streamlined the curriculum by identifying overlapping content and redistributing topics across other courses, optimizing the overall course sequence. This approach freed 2 SHs, which were then allocated to the new course without increasing the total credit requirement for students.

Next, we identified the most clinically relevant topics and designed the course accordingly. The greatest challenge was the limited number of SHs, as covering both recommended areas typically requires at least two separate courses—one in computer science and another in mathematics or statistics. To address this constraint, we implemented a novel approach to homework assignments, allowing us to effectively integrate both subject areas within a single course.

In this paper, we present our efforts in developing this new teaching approach that addresses the two recommendations from AAPM Report No. 365^1^ within a single course design. We provide an overview of the course content as offered in the Spring 2025 semester, and report students’ pre‐ and post‐course self‐assessments along with potential improvements for future iterations.

## 2 INNOVATION

### 2.1 Philosophical foundations of the new curriculum

The primary challenge in designing the course curriculum was the limited number of SHs available to cover two distinct topics. As noted above, the typical approach would involve students taking two separate courses—one offered by the computer science department and another by the mathematics or statistics department. However, for the reasons stated earlier, we considered this arrangement to be suboptimal, even if offering additional courses were feasible.

The ongoing and continuous evaluation of the program curriculum, most recently conducted in 2023, concluded that only a single 2‐SH course could be allocated. Recognizing that curriculum development is an iterative process, we carefully considered how best to maximize the educational impact within this constraint and decided to develop a new 2‐SH course, *MPHY 206: Mathematical and Computational Methods for Medical Physics*. In this new course, we adopted a novel approach toward homework assignments: rather than solving problems manually, students were required to write computer programs to complete most assignments. These carefully designed tasks not only reinforced their understanding of the course material but also promoted the practical application of computational methods and the development of coding skills.

### 2.2 Design framework

At our institution, the Provost's Office serves as the liaison among academic units, the institutional bulletins, and academic records, coordinating the development, review, and approval of curricula. The Provost's Office also periodically organizes workshops to support faculty in enhancing their teaching and curriculum design skills. Following its guidance, the College of Liberal Arts and Sciences (HCLS), under which our graduate medical physics program is housed, developed the “Guidelines for Syllabus Best Practices.”

The syllabus for this new course was developed following these guidelines, which specify several required components incorporated by the instructor during curriculum development. The sequence of development aligned with the well‐established backward design framework recommended by the Association for Supervision and Curriculum Development (ASCD).^3^

The backward design framework recommended by ASCD^3^ consists of three stages: (1) Identification of desired learning outcomes, (2) Determination of assessment evidence, and (3) Planning of instructional activities. In the following sections, we summarize the design framework for this new course using this three‐stage curriculum development process.

#### 2.2.1 STAGE 1: LEARNING OBJECTIVES

In the first stage, the learning objectives of the course were defined. Upon completion of the course, students are expected to:

1. Demonstrate an understanding of the mathematical and statistical foundations underlying radiation modeling, medical image processing, statistical methods, medical informatics, optimization, and introductory artificial intelligence (AI)/machine learning in medical physics as specified in AAPM Report No. 365^1^; and
2. Apply this knowledge to design and implement medical physics applications, thereby strengthening computational and programming skills.

#### 2.2.2 STAGE 2: ASSESSMENT STRATEGY

Assessment methods were selected to evaluate the achievement of the stated learning objectives. Evidence for assessment was primarily collected through:

1. Formative assessments: Coding‐based assignments.
2. Summative assessments: Midterm and final examinations.
3. Pre‐ and post‐self‐assessments.

Final grades were determined based on both formative and summative assessments. Self‐assessment results were solely utilized as references for future course improvement and development.

##### 2.2.2.1 Formative assessments

Following each lecture, students were assigned coding‐based homework as formative assignments. These assignments required the application of concepts introduced and discussed in class. Students were permitted to use a programming language of their choice. Additionally, the instructor implemented an open policy: students could reuse instructor‐provided, publicly available, or AI‐generated code, provided they thoroughly reviewed it, added explanatory comments, and modified it as needed to produce the required results. For each assignment, students were required to submit a report that included the developed code, resulting outputs, and a discussion demonstrating their understanding of the underlying concepts and their application to the assigned problem. Students had the option to resubmit their report if they were not satisfied with the grade received, revising their work in accordance with the instructor's feedback to potentially improve their grade.

##### 2.2.2.2 Summative assessments

Two examinations were administered during the course—one at midterm and one at the end of the term—as summative assessment to assess overall achievement of the course objectives. Each examination consisted of approximately 25% basic‐level questions, 50% intermediate‐level questions, and 25% advanced‐level questions. The basic and intermediate questions were designed to assess core competence across the class, while the advanced questions were intended to identify students demonstrating higher levels of mastery.

##### 2.2.2.3 Self‐assessments

Two self‐assessments—administered at the beginning and end of the course—were used to measure students’ improvement. Students were asked to rate their understanding of the topics covered during the semester using a 6‐point scale (0 to 5). The results of these self‐assessments were not used in the determination of students’ final grades.

#### 2.2.3 STAGE 3: INSTRUCTIONAL DESIGN

Instructional activities were designed to support effective knowledge delivery, conceptual understanding, and skill development. In this new course, the primary instructor implemented three strategies for each goal.

For knowledge delivery, foundational concepts were introduced through didactic lectures, which were supplemented by PowerPoint slides. The lecture content was carefully structured to fit within the allotted class time. To support student preparation, PDF versions of the slides were provided to students at least two days in advance.

Conceptual understanding was reinforced through in‐class questioning and discussions. These sessions included advanced questions and clinical examples that illustrated the practical applications of the material within medical physics contexts. Students were encouraged to answer these questions by drawing upon the lecture materials recently covered.

Skill development was primarily fostered through required homework assignments, which were structured as coding‐based tasks. These assignments required students to apply course concepts by solving problems with code, thereby reinforcing learning through practical implementation. To further assist students, supplementary sample code blocks were included in each homework assignment to illustrate the implementation of key Python functions relevant to the assigned tasks. Student knowledge acquisition was assessed based on the submitted assignment reports.

### 2.3 Topic selection

Rather than strictly following one or two textbooks, we selected topics based on the practical clinical and research needs of medical physicists. As all students in the program take the same core courses regardless of their subspecialty, the selected topics needed to be broadly relevant across all areas of medical physics rather than focused on only one or two.

To obtain diverse perspectives, we consulted imaging, therapy, and health physicists at our clinical affiliate institution—many of whom also serve as program faculty. This consultation was conducted as an informal survey through discussions during faculty and research meetings, as well as through a review of the course syllabi they teach, with the goal of identifying the essential mathematical and statistical topics most pertinent to their daily professional practice. Their expertise, combined with the primary instructor's (the first author's) more than 30 years of clinical and research experience, guided the development of a focused and highly relevant list of topics well suited for a one‐semester, 2‐SH course.

Once the topics were finalized, we reviewed a range of textbooks to identify relevant materials to serve as references. Although some content was drawn from medical physics texts, much of it was sourced from related disciplines such as statistics, mathematics, and psychology. For emerging areas such as machine learning, textbooks specifically tailored to medical physics were not yet available; therefore, we curated high‐quality online resources from reputable sources to supplement the course content. The new course was first launched in the Spring 2024 semester.

The course syllabus underwent several modifications during the Spring 2024 semester but has remained largely stable since the middle of the Spring 2025 offering. Table 1 presents the course outline for the Spring 2025 semester, along with the references consulted by the instructor in preparing the learning materials. The 2025 version shown in Table 1 is more than 95% identical to the final version used in 2024. The final curriculum covers key mathematical and statistical concepts, including signals and systems, Fourier series and transform, probability, statistical inference, image quality, optimization methods, and an introduction to AI with an emphasis on machine learning.

**TABLE 1:** Course outlines for the new *MPHY 206: Mathematical and Computational Methods for Medical Physics* offered in the Spring 2025 semester. References following each topic or subtopic indicate the sources the instructor referred to when preparing the learning materials, including handouts and homework assignments.

**Signals and systems (Lectures 1–2)** ^4^, ^5^, ^6^ Description of signals^4^, ^5^ Description of systems^4^, ^5^ Convolution^4^, ^5^ Discrete signal processing^4^ **^–^** ^6^ **Fourier transform (Lectures 3–5)** ^4^, ^6^, ^7^ Fourier series and Fourier transform^7^ Properties of the Fourier transform^4^, ^7^ Transfer function^4^, ^7^ Circular symmetry and the Hankel transform^4^ Discrete Fourier transform^4^, ^6^ Sampling theory and Nyquist criterion^4^, ^6^ **Probability and medical informatics (Lectures 6)** ^8^, ^9^ Gaussian, exponential, and Poisson distributions^8^ Random variable, independence^8^ Law of large number, central limit theory^8^ DICOM^9^ **Midterm exam**	**Statistical methods (Lectures 7–9)** ^8^, ^10^, ^11^ Likelihood function^8^ Point estimation^8^ Confidence interval^8^ Hypothesis testing^8^ Linear model^8^ Nonparametric methods^10^, ^11^ **Image quality (Lectures 10)** ^4^, ^12^, ^13^ Resolution^4^ Noise^4^ Signal‐to‐noise ratio^4^ Rose model^12^, ^13^ **Optimization methods (Lectures 11)** ^14^, ^15^, ^16^, ^17^, ^18^ System of linear equations^14^ Iterative technique^15^, ^16^ Algebraic reconstruction method^16^ Conjugate gradient method^17^ ML‐EM reconstruction method^15^, ^18^ **Artificial intelligence (Lectures 12)** ^19^, ^20^, ^21^, ^22^, ^23^, ^24^, ^25^, ^26^, ^27^ Introduction to machine learning^19^, ^20^, ^21^, ^22^ Support vector machine^23^, ^24^ AI in medical physics^25^, ^26^, ^27^ **Final exam**

#### 2.3.1 Illustrative example #1 — Fourier series and transform

**FIGURE 1:**
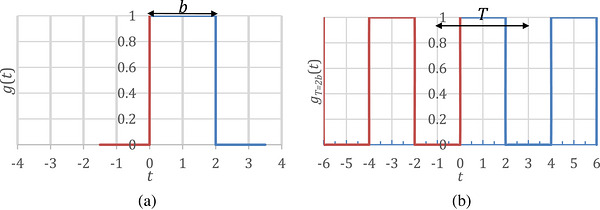
Example of a homework assignment for the new course. In this assignment, (a) g(t) is a rect function with full‐width b=2, and (b) $g_{T=2b}\left(t\right)$ is a periodic function of g(t) with period T. Students were asked to use the numerical integration to find the Fourier series coefficients $c_{m}$ of $g_{T=2b}\left(t\right)$ for m=−8,…,0,…,8, and perform Fourier transforms of these two functions for a specified range of integration, for example, for t=−10.25Tto10.75T.

Figure 1 provides an example of a homework assignment from the new course. In this problem, students were asked to use numerical integration to calculate the Fourier series coefficients $c_{m}$ of $g_{T=2b}\left(t\right)$ for m=8,…,0,…,8, perform Fourier transforms of these two functions for a specified range of integration, for example, for t=−10.25Tto10.75T, and compare the Fourier series coefficients with the Fourier transform spectrum.

**FIGURE 2:**
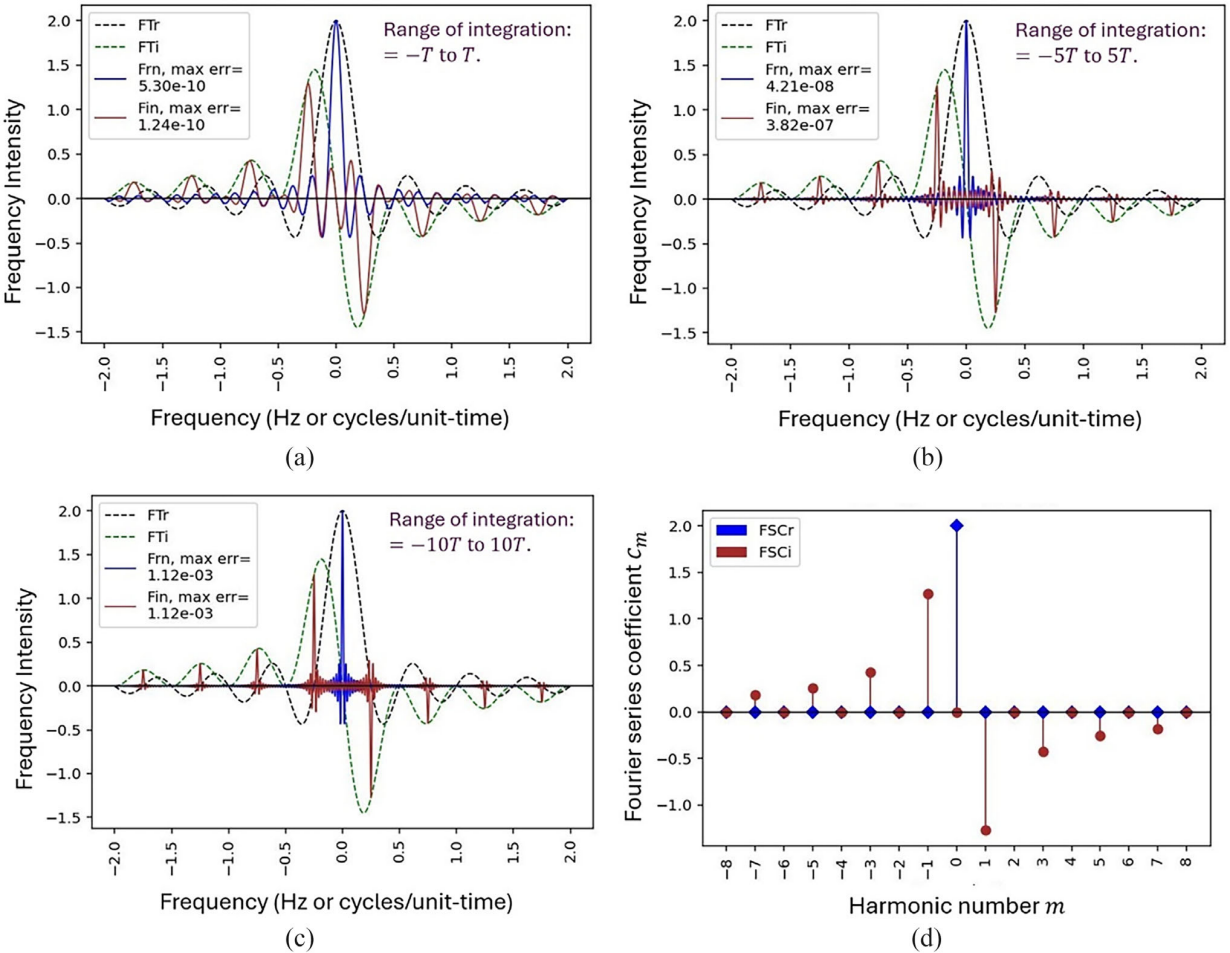
Results of the homework assignment in Figure 1. In panels (a)–(c), the dotted lines represent the real (blue) and imaginary (green) parts of the Fourier transform of g(t), while the solid lines show the real (blue) and imaginary (red) parts of the Fourier transforms of $g_{T=2b}\left(t\right)$ computed over three integration ranges: t=−1.25Tto1.75T, −5.25Tto5.75T, and −10.25Tto10.75T, respectively. Panel (d) shows the real (blue) and imaginary (red) parts of complex Fourier series coefficients $c_{m}$ of $g_{T=2b}\left(t\right)$. Since T=4 in Figure 1, each harmonic corresponds to a multiple m of the fundamental frequency $f_{0}=1/4=0.25$ Hz.

Figure 2 shows the expected results of the homework assignment in Figure 1. In panels (a)–(c), the dotted lines represent the real (blue) and imaginary (green) parts of the Fourier transform of g(t), while the solid lines show the real (blue) and imaginary (red) parts of the Fourier transforms of $g_{T=2b}\left(t\right)$ computed over three integration ranges: t=−1.25Tto1.75T, −5.25Tto5.75T, and −10.25Tto10.75T, respectively. Panel (d) shows the real (blue) and imaginary (red) parts of complex Fourier series coefficients $c_{m}$ of $g_{T=2b}\left(t\right)$. Since T=4 in Figure 1, each harmonic corresponds to a multiple m of the fundamental frequency $f_{0}=1/4=0.25$ Hz, which aligns with the peak locations in Figures 2a–c, although the alignment may be less apparent in Figure 2a.

The purpose of this question was to use numerical calculations and graphical illustrations to deepen students’ understanding of how the Fourier transform can be approximated by the Fourier series, as explained in the classroom lectures. Through this assignment, students gained an intuitive understanding that the Fourier transform of a periodic function like $g_{T=2b}\left(t\right)$ converges to its Fourier series coefficients as the integration range approaches (−∞,+∞)—a concept often challenging to grasp through traditional classroom instruction alone. Additionally, through this assignment, students observed the behavior of the Gibbs phenomenon as the integration range increases, offering deeper insight into another concept that is often difficult to understand through theoretical instruction alone.

#### 2.3.2 Illustrative example #2 — Shift property and sampling theory

**FIGURE 3:**
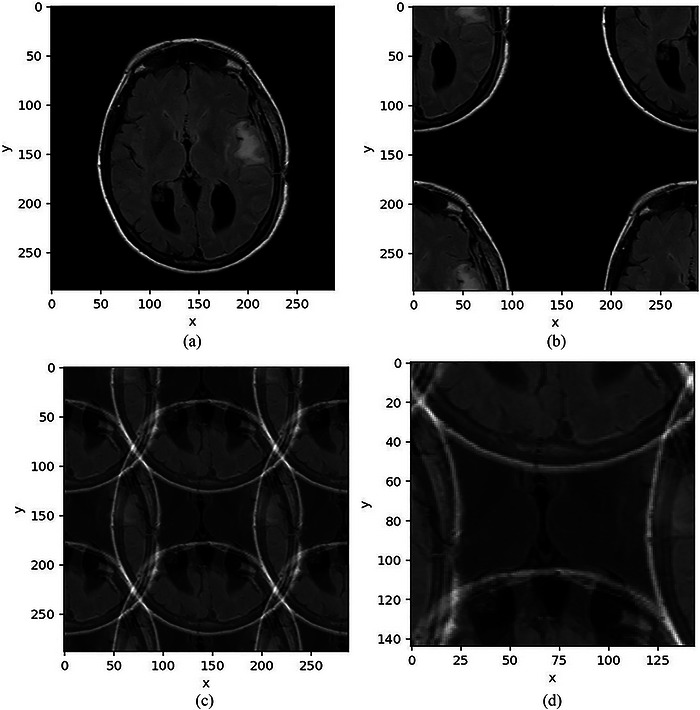
Example and results of another homework assignment for the sampling theory module of the new course. (a) Original MR brain image. (b) IDFT image obtained after applying a linear π‐radian phase shift in the frequency spectrum. (c) IDFT image reconstructed from a downsampled spectrum using the zero‐masking method. (d) IDFT image reconstructed from a downsampled spectrum using decimation and applying a linear π‐radian phase shift.

This homework assignment was specifically designed to help students develop a deeper understanding of several key properties of the discrete Fourier transform (DFT). In this exercise, students were asked to apply various modifications to the frequency spectrum of Figure 3a, a 288 × 288 MR brain image, and to observe the corresponding effects in the spatial domain.

Specifically, students were instructed to perform DFT on Figure 3a, apply a linear π‐radian phase shift by multiplying each pixel (u,v) in the frequency spectrum by $e^{-j(\pi u+\pi v)}$, and then perform the inverse DFT (IDFT) and display the result in the spatial domain. The expected result, shown in Figure 3b, corresponds to a half‐period shift of Figure 3a in both the x‐ and y‐directions. This exercise reinforced students’ understanding of the shift properties of the Fourier transform in both the spatial and frequency domains, as discussed in the classroom lecture.

Students were then asked to implement two downsampling approaches. The first was a zero‐masking (or replace‐with‐zero) method. In this approach, students downsampled the frequency spectrum by retaining only the even‐indexed pixels (2u,2v) and setting all other entries to zero.

This produced a matrix of the same size (288 × 288) as the original, with three‐quarters of its entries set to zero. The IDFT was then performed, and the expected result is shown in Figure 3c, which represents an aliased version of Figure 3a, with the spatial repetition period reduced by half due to the doubled frequency‐domain sampling interval.

The second approach was downsampling by decimation, in which students were instructed to retain only the even‐indexed pixels of the frequency spectrum in both the u and v directions, resulting in a matrix one quarter of the original size—that is, half the size in each dimension (144 × 144). A linear phase shift of $e^{-j(\pi u+\pi v)}$ was then applied to each pixel (u,v) of the reduced frequency spectrum, followed by the IDFT. Figure 3d shows the expected result of this exercise, which corresponds exactly to the central 144 × 144 crop of Figure 3c.

One concept students consistently struggled with was that the DFT assumes not only that the Fourier spectrum is periodic, but also that the input data array represents a single period of an infinitely repeating image in both directions of the spatial domain. Students typically developed a clearer understanding of this unintuitive concept after generating images similar to Figure 3c. This understanding was further reinforced when they realized that, by applying the shift property, they could select and crop any period of the overall image by introducing an appropriate linear phase shift, as demonstrated in Figure 3d.

Another challenging topic was sampling theory and aliasing. While it is generally easier to explain and illustrate how undersampling in the spatial domain leads to aliasing in the frequency domain, teaching the symmetrical property—where undersampling in the frequency domain produces aliasing in the spatial domain—proved more challenging to explain. Despite multiple classroom explanations and examples, students typically did not develop a solid understanding of this concept until they generated and observed artifacts similar to those in Figures 3c,d. A strong grasp of spatial‐domain aliasing also equips students to communicate effectively with the radiology department when encountering aliased MR images.

### 2.4 Coding requirements

Students could choose the programming language they were most comfortable with but were encouraged to use modern, structured, languages such as MATLAB, C++, Java, R, or Python—many of which support object‐oriented programming and all of which offer extensive libraries relevant to the topics covered in this course. Students at our institution have free access to MATLAB, including all libraries essential for the course, while C++, Java, R, and Python are freely available as open‐source or publicly accessible programming languages.

For each coding‐based assignment, students were required to submit a comprehensive report including:

1. A clear explanation of their logical reasoning,
2. The complete, runnable code,
3. The results (including figures or outputs), and
4. A brief discussion of their findings.

In the past two offerings (Spring 2024 and 2025), most students submitted their coding reports on time, while those who missed the original deadline typically completed their submissions within one to two weeks. The mean score for all coding‐based assignments was approximately 8.9 ± 1.5 on a scale of 0 to 10, indicating that students generally produced satisfactory code and results and could reasonably interpret the mathematical and statistical concepts they were implementing. A small number of students who struggled with coding were referred to our institution's tutoring resources and received additional support.

### 2.5 Self‐assessment of the new course

The new 2‐SH in‐person course, *MPHY 206: Mathematical and Computational Methods for Medical Physics*, was offered in the Spring 2024 and 2025 semesters to first‐year students in the MS in Medical Physics program at our institution. During the second offering (Spring 2025 semester), the nine enrolled students completed a self‐assessment at the beginning and end of the semester.

In this assessment, students rated their understanding of 23 course elements on a 6‐point scale (0 to 5), with 5 indicating the highest level of understanding or competency. The instrument used for this self‐assessment is provided in Appendix A. The purpose of this assessment was to evaluate students’ grasp of the course topics and improvements in coding skills, rather than to grade their performance. Results were analyzed using a two‐tailed paired *t*‐test with a 5% significance level.

Table 2 presents the self‐assessment results from the nine students enrolled in the Spring 2025 semester. Results from the paired *t*‐test indicate that the improvements were statistically significant (*α* < 0.05) for all course elements (as shown in the “Diff_mean_” column) except for the Probability element (*α* = 0.0516), as well as for the overall average improvement (mean of Diff_mean_, reported at the bottom of the table).

**TABLE 2:** Self‐assessment results from nine students enrolled in the Spring 2025 semester. Students rated their understanding of 23 course elements on a 6‐point scale (0 to 5) at the beginning and end of the course. Two‐tailed paired *t*‐tests were used to evaluate score improvement (*α* = 0.05), both for each individual element (as shown in the “Diff_mean_” column) and for the overall improvement (calculated as the mean of the Diff_mean_ values, shown at the bottom of the table).

#	Course elements (Scale 0–5 with 5 as best)	Mean_pre_	Mean_post_	Diff_mean_	Diff_STD_	*p*‐value	Stat sig
1	Familiarity with the programming language	2.56	3.83	1.28	0.75	0.0010	Y
2	Signals and systems	1.44	3.33	1.89	0.78	0.0001	Y
3	Fourier transform	1.44	3.11	1.67	0.71	0.0001	Y
4	Probability	2.89	3.78	0.89	1.17	0.0516	N
5	Statistical inference	2.00	3.44	1.44	1.13	0.0050	Y
6	Image quality	1.89	3.44	1.56	1.01	0.0017	Y
7	Optimization methods	1.22	3.00	1.78	0.97	0.0006	Y
8	Machine learning	1.33	2.67	1.33	1.12	0.0072	Y
9	Data visualization	3.33	4.11	0.78	0.97	0.0431	Y
10	Numerical integration	3.11	3.78	0.67	0.87	0.0497	Y
11	Convolution	0.89	3.22	2.33	1.00	0.0001	Y
12	Continuous‐time, discrete‐time, and fast Fourier transforms	1.22	3.00	1.78	0.97	0.0006	Y
13	Random number generator	3.00	4.44	1.44	1.01	0.0027	Y
14	Point estimation	2.11	3.89	1.78	1.20	0.0022	Y
15	Confidence interval	2.33	3.78	1.44	1.74	0.0375	Y
16	Hypothesis testing	1.89	3.89	2.00	1.50	0.0039	Y
17	Linear models	2.11	3.89	1.78	1.09	0.0012	Y
18	DICOM	1.89	3.56	1.67	1.66	0.0167	Y
19	Rose model	0.67	2.89	2.22	1.09	0.0003	Y
20	Conjugate‐gradient descent method	0.78	2.44	1.67	1.00	0.0011	Y
21	Iterative methods for solving systems of linear equations	1.33	2.89	1.56	1.13	0.0033	Y
22	Support vector machine	1.00	2.33	1.33	1.22	0.0114	Y
23	Applications of AI in medical physics	1.00	2.67	1.67	1.32	0.0054	Y

*Note*: Mean of Diff_mean _= 1.56, STD of Diff_mean_ = 0.41, p‐value = 7.68E‐15, Stat sig: Y.

In addition, results not shown in Table 2 represent the average improvement across all 23 items for individual students. Every student demonstrated statistically significant gains, with p‐values several orders of magnitude smaller than 0.05—the largest observed *p*‐value was 0.0011.

It is noted that Table 1 lists 31 subtopics, but only 23 course elements were evaluated in Table 2. This discrepancy occurred because the course outlines in Table 1 tend to be more detailed, while the evaluation elements in Table 2 may combine multiple subtopics from Table 1. Additionally, the evaluation elements had to be defined at the beginning of the semester, whereas the exact course content could be adjusted during the semester to better meet students’ needs.

For example, there were six subtopics under Statistical Methods (Lectures 7–9) in Table 1, but only four corresponding elements were evaluated in Table 2. This is because the “likelihood function” serves as foundational background for the subsequent statistical methods and is therefore not evaluated separately. The topic of “nonparametric methods” was added during the semester after being identified as important, but it was introduced too late to be included as an assessment element in Table 2.

Finally, our institution administered student evaluations for each course at the end of each semester, culminating in a Course and Teacher Rating (CTR) report. This survey assesses 16 individual items related to the quality of the course and instructor. Due to confidentiality requirements, detailed results are accessible only to instructors, faculty members, and the department and therefore cannot be disclosed in this publication. However, an analysis of the past two years' CTR reports for this new course revealed that while most students found it educationally beneficial, they consistently reported a high level of difficulty. Specifically, among the four measures used to assess workload and difficulty (“Pace of Course,” “Difficulty,” “Reading Material,” and “Exam/Paper Demand”), students indicated that the “Exam/Paper Demand” category was the most challenging. Given that coding‐based assignments constituted the primary workload for this course, we interpret this as students reporting a high level of difficulty primarily with these assignments.

## 3 DISCUSSION

The goal of this new course was to cover the two topics recommended in AAPM Report No. 365^1^: (1) Mathematical and statistical methods as well as (2) Computational methods and medical informatics—within a single 2‐SH course, avoiding an increase in the total SHs required for graduation. This goal was largely achieved, as evidenced by the self‐assessment results (Table 2). Regardless of their prior computational background, all students successfully developed or improved their coding skills and demonstrated a better understanding across the course elements listed in Table 1. All improvements were statistically significant, except for one element, “Probability,” which showed an improvement slightly above the threshold for statistical significance (*p* = 0.0516).

To achieve this, the course adopted an innovative approach in which students wrote computer programs to solve homework problems, rather than completing them manually. This unique strategy addressed the primary challenge of limited SHs allocated to cover both topics—mathematical and statistical methods, as well as computational methods and medical informatics—within a single course design.

Some may raise the concern that allowing students to use any programming language could complicate evaluation for the instructor. However, this did not pose a significant issue, as most structured programming languages share similar syntax, and the instructor is well‐versed in the major languages commonly used in the medical physics profession. Moreover, practical options available to students in the current time are largely limited to three languages: Python, R, and MATLAB—the first two being open‐source and MATLAB provided under an institutional license. As a result, the instructor was able to run any student‐submitted code whenever questions arose about its functionality.

Academic integrity, particularly regarding AI‐assisted coding, was another major concern. The use of AI‐generated or publicly available resources for completing homework assignments has become a complex and controversial issue across all levels of education, especially in programs where many assignments require written reports. At our institution, this topic has been a recurring point of discussion in faculty meetings over the past few years. As mentioned earlier, the instructor of this course adopted an open policy: students are permitted to reuse instructor‐provided, publicly available or AI‐generated code provided they review the acquired code thoroughly, add explanatory comments, and modify it to produce the required results. With a relatively small class size (approximately 10 students), the instructor was able to cross‐compare submissions and, over the past two course offerings, identified several instances in which code had been irresponsibly shared among students without significant modifications.

A legitimate question may arise as to whether a “two topics in a single course” approach is an effective teaching strategy for students who are not already proficient in mathematical and statistical concepts or in coding. This concern was carefully considered during the curriculum design. In practice, it is not feasible to teach all prerequisite material from first principles; therefore, a certain level of prior knowledge must be assumed. All admitted students meet CAMPEP admission requirements, which include coursework equivalent to a minor in physics. Consequently, students are not encountering the mathematical and statistical foundations of this course for the first time.

Instruction in coding presents a more nuanced challenge, as CAMPEP does not explicitly specify undergraduate programming requirements. Initially, we assumed that students entering the program would possess basic coding skills based on their STEM backgrounds. However, several years prior to offering this course, we recognized that this assumption was not consistently valid. While some students arrived with substantial programming experience, others had very limited exposure. CTR results further indicated that although students found the course educationally valuable, some reported significant difficulty with the coding‐based assignments.

A long‐term solution would be to require at least one introductory computer science course and one programming language course as part of the undergraduate STEM curriculum. However, such curricular requirements are beyond our control. As an interim measure, the welcome package for our medical physics graduate program, sent to admitted students in late spring or early summer, advises them to begin learning Python through self‐guided online resources prior to enrollment at our institution.

In addition, *MPHY 214: Radiological Physics and Dosimetry*—offered in the fall semester preceding this course—includes introductory‐level programming exercises. Students are required to write code simulating radioactive decay using a random number generator for the exponential distribution. Through this exercise, they explore counting statistics in health physics, demonstrating that the number of radioactive decays occurring within a fixed time interval follows a Poisson distribution. These assignments are intentionally straightforward and do not require extensive prior coding experience. Although not an intended goal, they effectively provide an informal introduction to programming for students with limited coding backgrounds.

Following the initial offering of the course in Spring 2024, supplementary sample code blocks were incorporated into each homework assignment for Spring 2025. These examples illustrate the implementation of key Python functions relevant to the assignments. Students are permitted to incorporate these code blocks into their work, provided they demonstrate a clear understanding of the purpose and functionality of each component.

As a result of these measures, most students enter the course with at least a foundational level of proficiency in both the necessary mathematical and statistical concepts and introductory programming. While individual strengths may vary, no student begins the course without prior exposure to either domain. With this preparatory structure—and assuming adequate background in mathematical physics or engineering mathematics—it is reasonable to expect students to write computer programs to complete the majority of course assignments.

Another concern regarding the “two topics in a single course” approach is whether the course design allows instructors to distinguish between deficiencies in conceptual understanding and difficulties with coding. This concern is addressed through the implementation of appropriate and distinct assessment strategies for each component. In the subsection “Stage 2. Assessment Strategy” of the Design Framework, we describe how students are evaluated with respect to the stated learning objectives.

In particular, reports for coding assignments are required to include the developed code, the resulting outputs, and a written discussion demonstrating students’ understanding of the underlying concepts and their application to the assigned problem. This structure enables the instructor to evaluate conceptual learning and coding proficiency separately. In addition, the midterm and final examinations provide further opportunities to assess students’ overall achievement of the course objectives. Therefore, we are confident that the course assessment framework allows us to distinguish between failures of conceptual learning and failures of coding, and to provide targeted support to students when difficulties arise.

Based on the success of this course and others (e.g., MPHY 214 mentioned above), we concluded that integrating coding into homework assignments significantly enhances students’ practical skills and deepens their understanding of complex concepts in clinical and research medical physics applications. Consequently, instructors throughout our program have been encouraged to increase the proportion of homework assignments and exam questions that require computer programming. For other courses, instructors are encouraged to assign coding tasks only when coding represents the most effective way for students to learn a particular topic. For example, no coding assignment was given for the Bragg‐Gray cavity theory in MPHY 214, as we did not believe such an assignment would enhance students’ understanding of the theory. However, we observed that students learned certain concepts—such as counting statistics and the Poisson distribution—more effectively by using a random number generator to simulate radioactive decay than through extensive in‐class lecturing alone. This approach represents a meaningful shift in how homework assignments can be designed—not only in medical physics graduate courses but potentially across other STEM disciplines.

One might reasonably ask, given the increasing demand for computational and AI‐based applications, how long the “two topics in a single course” approach can remain viable before additional courses—and their associated financial implications—become necessary. This question is inherently speculative and therefore difficult to address within the scope of this paper. Accordingly, the manuscript focuses on our current efforts to meet existing programmatic requirements, and we believe that the present curriculum represents a carefully considered and appropriate solution within these constraints.

Regarding the effectiveness of this course in preparing students for their careers, we cannot fully assess this outcome until our graduates begin working in medical physics. The first cohort to complete this course is currently in residency training, so their perspectives on how well the course has prepared them for professional practice are not yet fully available. Nevertheless, taking proactive steps is preferable to inaction. As the field of medical physics continues to integrate more information technology, this course provides a solid starting point and a foundation for learning and improvement in future offerings of similar courses.

With respect to future developments, the curriculum is subject to ongoing review and revision to ensure continued alignment with CAMPEP requirements and, more importantly, with the evolving educational and professional needs of the medical physics field. We are confident that this iterative, outcomes‐driven approach to curriculum review will enable the program to respond effectively and responsibly to future changes as they arise.

In conclusion, we have successfully developed and implemented a curriculum integrating both mathematical and statistical methods as well as computational methods and medical informatics components recommended in AAPM Report No. 365^1^. By requiring students to write computer programs to solve homework problems, we effectively covered both topics within a 2‐SH course without increasing the graduation credits. All measured student improvements were statistically significant. Future efforts will focus on providing more structured coding guidelines and incorporating emerging topics as they become relevant. This innovative approach may influence homework design in medical physics graduate courses and other interdisciplinary programs combining computational skills with theoretical or applied science and technology.

## AUTHOR CONTRIBUTIONS

Jenghwa Chang and Marissa Joyce Vaccarelli jointly restructured *MPHY 212: Foundations of Clinical Medical Physics* into two separate courses— *MPHY 206: Mathematical and Computational Methods for Medical Physics* and *M* *PHY 202: Ethical Conduct and Professionalism in Medical Physics*—for the M.S. in Medical Physics program at Hofstra University. Jenghwa Chang served as the primary instructor for MPHY 206 and developed the initial course syllabus. Marissa Joyce Vaccarelli contributed to the course content for MPHY 206, provided guidance on survey administration, and assisted with data analysis. Both Jenghwa Chang and Marissa Joyce Vaccarelli contributed to the writing and editing of this manuscript.

## CONFLICT OF INTEREST STATEMENT

The authors declare no conflicts of interest.

## APPENDIX A INSTRUMENT FOR SELF‐ASSESSMENT

Appendix A lists the instrument used for the self‐assessment of this new course in the Spring 2025 semester. It includes the assessment instructions, the complete set of 23 evaluation elements and the criteria defining each of the six levels of understanding.

### A.1 INSTRUCTIONS

Please evaluate your level of understanding for each of the following 23 items using the provided 6‐point scale (0 to 5) understanding levels:

1. Familiarity with the programming language
2. Signals and systems
3. Fourier transform
4. Probability
5. Statistical inference
6. Image quality
7. Optimization methods
8. Machine learning
9. Data visualization
10. Numerical integration
11. Convolution
12. Continuous‐time, discrete‐time, and fast Fourier transforms
13. Random number generator
14. Point estimation
15. Confidence interval
16. Hypothesis testing
17. Linear models
18. DICOM
19. Rose model
20. Conjugate‐gradient descent method
21. Iterative methods for solving systems of linear equations
22. Support vector machine
23. Applications of AI in medical physics

### LEVELS (6‐POINT SCALE)

0. No understanding (I never learned this before).
1. Little understanding (I start to understand but am still very confused).
2. Moderate understanding (I can do this with help or an example to look at).
3. Understand (I can do this on my own but might get confused once in a while).
4. Good understanding (I can confidently do this).
5. Very good understanding (I can teach).
